# There is Diversity in Disorder—“In all Chaos there is a Cosmos, in all Disorder a Secret Order”

**DOI:** 10.3389/fmolb.2016.00004

**Published:** 2016-02-11

**Authors:** Jakob T. Nielsen, Frans A. A. Mulder

**Affiliations:** Department of Chemistry and Interdisciplinary Nanoscience Center, University of AarhusAarhus, Denmark

**Keywords:** intrinsically disordered proteins, NMR spectroscopy, data interpretation, statistical, chemical shift, protein conformation

## Abstract

The protein universe consists of a continuum of structures ranging from full order to complete disorder. As the structured part of the proteome has been intensively studied, stably folded proteins are increasingly well documented and understood. However, proteins that are fully, or in large part, disordered are much less well characterized. Here we collected NMR chemical shifts in a small database for 117 protein sequences that are known to contain disorder. We demonstrate that NMR chemical shift data can be brought to bear as an exquisite judge of protein disorder at the residue level, and help in validation. With the help of secondary chemical shift analysis we demonstrate that the proteins in the database span the full spectrum of disorder, but still, largely segregate into two classes; disordered with small segments of order scattered along the sequence, and structured with small segments of disorder inserted between the different structured regions. A detailed analysis reveals that the distribution of order/disorder along the sequence shows a complex and asymmetric distribution, that is highly protein-dependent. Access to ratified training data further suggests an avenue to improving prediction of disorder from sequence.

## Introduction

A systematic re-examination of the protein structure–function paradigm is required to accommodate intrinsically unfolded/disordered proteins (IDPs). There are two major reasons for this reappraisal: (1) the results of bioinformatics analyses of the genomic codes for protein amino acid sequences, and (2) the accumulation of experimental evidence for the existence of a rather large number of protein domains and even entire proteins, lacking ordered structure under physiological conditions (Dyson and Wright, 2001, 2004; Uversky, 2003; Vucetic et al., 2003). Analysis of sequence data for complete genomes indicates that intrinsically disordered proteins are highly prevalent, and that the proportion of proteins that contain such segments increases with the increasing complexity of an organism; Putative long (>30 residue) disordered segments are found to occur in 2.0% of archaean, 4.2% of eubacterial, and 33% of eukaryotic proteins (Ward et al., 2004). Protein disorder roughly segregates into three major classes, depending on whether disorder serves a primary functional role, or serves permanent or transient interactions (van der Lee et al., 2014). Moreover, disorder maps to proteins with important functions, such as signal transduction and control of transcription, and IDPs are involved in all major classes of disease (Gregersen et al., 2006; Uversky et al., 2008). Although proteins may adopt different structures inside a cellular milieu, this paper is concerned solely with proteins studied under *in vitro* conditions. For the biological relevance of disorder for the selected proteins, the interested reader is referred to the original papers that described the proteins considered herein.

This paper draws on methodological advances in NMR spectroscopy to study IDPs, and systematic analysis of chemical shift data for the prediction of disorder from sequence. The aim of this work is to develop an experimentally calibrated “ruler” to detect and quantify sequence-specific protein disorder. NMR chemical shifts offer highly reliable and redundant residue-specific information on positional disorder, and this information is easy and unambiguous to get, using recently developed approaches (Jensen et al., 2013; Kragelj et al., 2013; Felli and Pierattelli, 2014; Konrat, 2014). In addition, the growing amount of NMR chemical shift assignment data now allows for rigorous and comprehensive analysis of protein disorder, and to employ this ruler to gauge the types of variation of protein disorder.

In this paper, we search for any potential trends or variations in order/disorder in an assorted set of proteins. To this end, we constructed a comprehensive collection of proteins with varying degrees of (partial) disorder, for which assigned NMR chemical shifts are available. We subsequently asked: “Is disorder similar in proteins, or are there different patterns to be discerned?,” “What is a *typical* variation between order/disorder?,” and “Are there proteins that deserve the label *super unfolded*, and are they representative of the general class of IDPs?.” We demonstrate that it is possible to answer these questions, with the methods discussed herein. It is hoped that this initial experimental evaluation of residue-specific protein positional disorder will spark the further evolution of assessment tools for predicting disorder with greater detail and accuracy. In addition, our analysis paints a validated picture of protein disorder for a diverse subset of 117 example proteins that are either classified as IDPs, or possess long intrinsically disordered regions (IDRs), showing a highly abounding sequence context dependence.

## Methods

### Generation of a curated database with available NMR chemical shift data

A set of proteins with different degrees of structural disorder, for which assigned chemical shifts were available, was generated in two steps. First, a set of proteins was generated from a keyword search in the BioMagResBank (BMRB) database (http://www.bmrb.wisc.edu; Ulrich et al., 2008). Second, we augmented this database with sequences from the Database of Protein Disorder (DisProt) (http://www.disprot.org; Vucetic et al., 2005; Sickmeier et al., 2007) of disordered regions, for which data were also present in the BMRB. In the first step, the BMRB database was searched for entries according to the BMRB database tag for the physical state of the protein; Entries were selected where _Entity_assembly.Physical_state = “denatured,” “intrinsically disordered,” “molten globule,” “partially disordered,” and “unfolded.” In addition, entries with the words “disordered” or “unstructured” in the entry title were also included. In the second case, the BMRB database was searched for matches of all SwissProt identifiers present in the DisProt database. The sequences from DisProt and BMRB were aligned using the EMBOSS (http://www.ebi.ac.uk/Tools/psa/emboss_needle/) implementation of the Needleman–Wunsch alignment algorithm (Needleman and Wunsch, 1970) and BMRB entries with >20% of the aligned residues classified as disordered by DisProt were retained. In addition, an entry with in-house assigned chemical shift for the N-terminal heavy metal binding domain, residues 1–84, of a cupper-binding ATP-ase (Gourdon et al., 2011) in an unstructured state was added to the database.

Subsequently, the database was curated. First, conditions that deliberately destabilize folded proteins, such as extremes of pH, extremes of temperature, and denaturants were selected against, in order to avoid the artificial inclusion of disorder under non-native conditions. This step also removes any digressions that such conditions have on the chemical shifts: Only proteins with near-neutral pH (4 < pH < 9), in weak salt buffer, not in complex with other molecules, and without modified amino acids were kept. Typical conditions of excluded entries were: pH < 4.0, bicelle/micelle/SDS present in buffer, TFE/DMSO/GuHCl or other denaturants added, presence of co-factors, and phosphorylation. Next, entries having fewer than 40 amino acid residues or fewer than 50 assigned chemical shifts were removed. Third, the collection of entries was culled in two steps to remove redundant data. To find highly similar entries, the EMBOSS Needle program was used to calculate pairwise sequence identity between the sequences. In the first step, sets of proteins families, defined as sets of chains with >90% mutual sequence identity, were identified and only the entry with the most native-like sequence/condition from each set was kept in the database. For example, wild type sequences were kept and mutant sequences were discarded when both were present, and in case data were available at different acidities, the pH closest to neutral was preferred. In the second step, groups of homologous chains, defined by having >50% sequence identity between at least one other sequence in the set, were identified. In each set, the subset of sequences that yielded the maximum total number of chemical shifts, and where all pairs had < 50% sequence identity, were kept. Finally, it was required that at least one residue could be defined as disordered based on NMR chemical shift data (see below, Equation 5). This procedure resulted in the construction of a set of 117 proteins with assigned chemical shifts, with varying degrees of structure/disorder. The list of 117 BMRB identifiers is provided as Table S1.

### Calculation of order/disorder metrics from experimental NMR data

Weighted sum of squared differences between observed and predicted shifts were calculated as follows

(1) $$\begin{array}{r} \chi^{2}\left(i\right)=\underset{n}{\sum }\underset{j=i-1,i,i+1}{\sum }\text{min}\left({\Delta_{jn}}^{2},16\right) \end{array}$$

where the difference was truncated to four standard deviations using:

(2) $$\begin{array}{r} \Delta_{jn}=\frac{\delta_{obs}\left(j,n\right)-\delta_{pred}\left(j,n\right)}{\sigma \left(n\right)} \end{array}$$

Here δ_*obs*_(*j, n*) is the observed (offset corrected) chemical shift of atom type *n* for residue *j*, and δ_*pred*_(*j, n*) is the neighbor corrected random coil chemical shift predicted based on the tripeptide centered at residue *j* (Tamiola et al., 2010). The chemical shift difference is scaled with the expected difference for residue *j* in an IDP using σ(*n*) = 0.627, 0.310, 0.219, 0.281, 0.102, 0.0566, 0.0546 ppm for *n* = N, C′, Cβ, Cα, H_N_, Hα, and Hβ, respectively. The supposed chi-square distributed number χ^2^ is transformed to an approximately normal distributed number *L*, by using linear combinations of fractional powers of χ^2^(Canal, 2005).

(3) $$\begin{array}{r} L=\rho^{1∕6}-\frac{1}{2}\rho^{1∕3}+\frac{1}{3}\rho^{1∕2},\;\rho =\frac{\chi^{2}}{N} \end{array}$$

where *N* is the number of assigned chemical shifts for the triplet. *L* is converted to a standard normal distributed number, *Z*_*IDR*_, by correcting with the known mean and standard deviation for *L* (Canal, 2005).

(4) $$\begin{array}{r} Z_{IDR}=\frac{L-\mu_{L}\left(N\right)}{\sigma_{L}\left(N\right)} \end{array}$$

We assign local sequence specific residue states as either *disordered* (D) according to the definition:

(5) $$\begin{array}{r} Z_{IDR}<3 \end{array}$$

or alternatively as *ordered* (O) if *Z*_*IDR*_ ≥ 3. A protein sequence can be thought of as consisting of alternating segments of disordered and ordered residues with lengths *s*_*i*_. The *sequence disorder complexity*, *C*_*SD*_, is now defined as

(6) $$\begin{array}{r} C_{SD}=\frac{e^{H}-1}{N},\;H=-\underset{i}{\sum }\frac{s_{i}}{N}\text{ln}\left(\frac{s_{i}}{N}\right), \end{array}$$

where *N* is the number of residues in the protein. Note that *H* is closely related to the Shannon entropy of a statistical distribution. If a protein is built of *n* segments of equal length then *e*^*H*^ = *n* and if the segments have different lengths *e*^*H*^ < *n*. In particular, a protein with exclusively disordered/ordered residues (pure state) will have *H* = 0, *e*^*H*^ = 1, and *C*_*SD*_ = 0 whereas a protein where order and disorder continuously alternate along the sequence has a maximal value of *C*_*SD*_ = 1. If order and disorder are independently randomly distributed with a probability of 0.5, we simulated with a random number generator that the sequence disorder complexity would be ca. 0.41 on average. For a general probability, *p*, and random outcome: *C*_SD_ ≈ *p*. Therefore, it make sense to compare the sequence disorder complexity to the fraction of disordered residues *f*_D_ = *N*_D_/*N* where *N*_D_ is the number of disordered residues (*Z*_*IDR*_ < 3). As such, the *relative sequence disorder complexity*, *C*_SD_/*f*_*D*_ is expected to have a smaller variation.

### Procedure for re-referencing assigned chemical shifts

Chemical shifts are deposited using different referencing procedures at different conditions such as temperature, added salt, and pH, and hence, it is likely that in some cases the observed chemical shift would be slightly, yet systematically, offset from the random chemical shift derived from the sequence. However, since even small deviations from random coil shifts are indicative of structure ordering, we estimated an offset correction for each entry in our database. The chemical shifts were re-referenced for each atom type independently using the following procedure: First, the neighbor corrected random coil chemical shifts were calculated for all residues following the procedure of Tamiola et al. as implemented in the program ncIDP (http://www.protein-nmr.org/; Tamiola et al., 2010), and the deviations from random coil chemical shifts, Δ, were calculated using Equations (1) and (2) above. Assuming that the NMR data is correctly referenced, this procedure identifies small deviations due to deviations in pH and temperature of the experimental data relative to the reference database. Next, the standard deviation of Δ was calculated for nine consecutive residues, and the sequence position with the smallest standard deviation was identified. The average of Δ for the nine residues was then used as candidate offset correction. The average value of *Z*_*IDR*_ was evaluated using (i) the candidate offset correction as described above and (ii) no offset correction. The scenario leading to the smallest average *Z*_*IDR*_ was chosen as the initial offset estimation (i.e., either using the candidate offset or no offset correction). because chemical shift distributions in IDRs show a distribution (Tamiola et al., 2010), much narrower than those in structured parts (Wang and Jardetzky, 2002; Zhang et al., 2003), owing to the many additional contributions to the chemical shifts in structured proteins (Wishart and Case, 2001; Shen and Bax, 2010; Nielsen et al., 2012). Finally, using the chosen initial offset estimation, the average Δ using all the chemical shifts for the particular atom type in disordered residues only (Equation 5) was calculated and this number was used as the final offset correction. To avoid eliminating true deviations originating from structure, in both cases, the revised offset was only used if there was a significant reduction in the chemical shift standard deviation, σ(δ_off_), relative to the uncorrected equivalent, σ(δ_0_), considering the number of chemical shift observations, *N*, by the application of Akaikes Information Criterion (Akaike, 1974, 1985) using a variance inflation factor of 10.0. i.e., the offset correction was only accepted if: N^*^ln(σ(δ_0_)/σ(δ_off_)) > 20.0 and *N* > 3.

## Results

### Analysis of chemical shift dispersion for seven case story proteins

**Figure 1:**
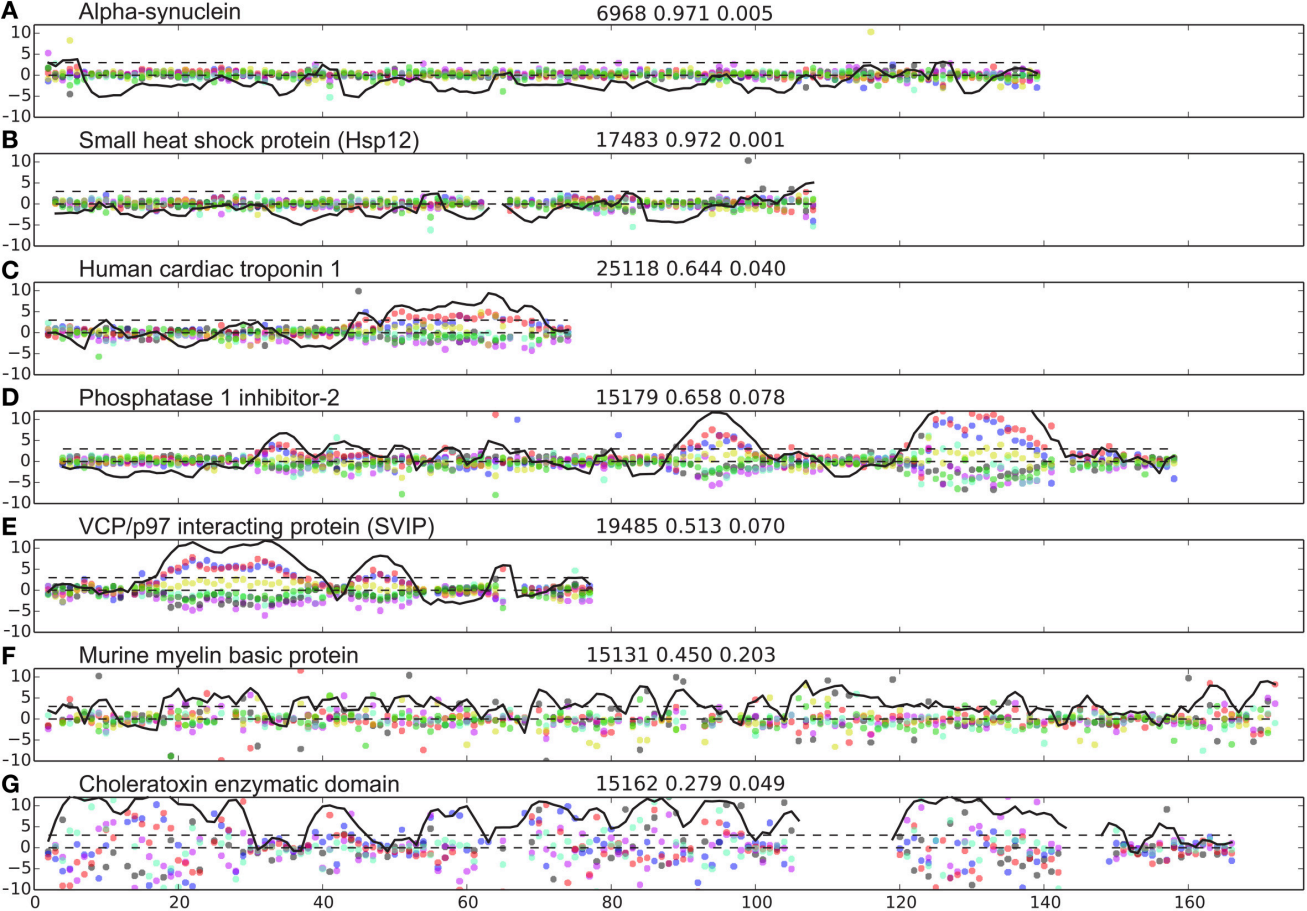
**Examples of IDP disorder profiles. (A–G)** weighted difference between observed and predicted shifts, Δ_jn_ (Equation 2) shown with blue, red, black, green, cyan, magenta, and yellow dots for C′, Cα, Cβ, Hα, H_N_, N, and Hβ, respectively. The Z-score (Equation 4) is shown as a black line, showing the lines, *Z* = 0 and *Z* = 3 for reference with black broken lines. The name of the protein analyzed is indicated at the top of each panel to the left. Three numbers are provided on top of each panel (middle) referring to, the BMRB id, the fraction of disordered residues, and the sequence disorder complexity, respectively.

The database of proteins containing disordered regions was constructed as described in Methods. This carefully curated database contains 117 unique proteins chains and 13,069 residues with 65,574 assigned backbone and Cβ/Hβ chemical shifts in total (excluding terminal residues). The database contains many well-characterized IDPs such as α-synuclein (αSyn, bmrbID = 6968) (Bermel et al., 2006), small heat shock protein (Hsp12, bmrbID = 17483; Singarapu et al., 2011), the CD79a cytosolic domain (bmrbID 18867; Isaksson et al., 2013), apo-IscU (bmrbID = 17836; Kim et al., 2012), and the cytoplasmic domain of human neuroligin-3 (bmrbID = 17290; Wood et al., 2012). For each protein in the database we calculated the scaled difference, Δ, from random coil shifts (Equation 2). This difference is plotted as a function of residue number for seven representative proteins from the database in Figure 1. It is seen that for the two proteins, known to be intrinsically unstructured, αSyn (bmrbID = 6968) and Hsp12 (bmrbID = 17483), fluctuations away from random coil shifts are very small throughout the sequence. This is also borne out by the fraction of disordered residues being 97% in both cases (see Methods Equations 1–5 and below). Conversely, two other proteins reported to be IDPs, the 18.5 kDa isoform of murine myelin basic protein (bmrbID = 15131; Libich et al., 2007), and the Cholera Toxin Enzymatic Domain (1–167) (bmrbID = 15162; Ampapathi et al., 2008) display larger scatter. Values for the fraction of disordered residues *f*_*D*_ are 0.45 and 0.28, respectively, which indicates that these order/disorder transitions of the cholera toxin enzymatic domain are essential for function, the protein is, in fact, mostly folded. For three further proteins, we note yet another pattern in their chemical shift residuals along the sequence, containing distinct separate segments with larger local spread of the chemical shifts for all nuclei. The examples chosen here are human cardiac troponin I, residues 1–73 (Hwang et al., 2014), inhibitor-2 involved in protein phosphatase 1 regulation (Kelker et al., 2007), and the small VCP/p97-interacting protein (Wu et al., 2013; BMRB ids: 25118, 15179, and 19485, respectively). In these examples, the larger local scatter is biased in the direction of downfield shifted C′ and Cα chemical shifts and upfield shifted Cβ, Hα, H_N_, and N chemical shifts (Figures 1C–E). This observation is consistent with the presence of helical structure formation. In addition, in these three examples, we see a variation in helix size within the same protein, as well as amongst different proteins. A variation in the amplitude of the chemical shift residuals is also observed. Since chemical shifts are time-averaged observables, smaller amplitude of the chemical shift residuals correspond to fractional occupancies of helical states as discussed previously (Marsh et al., 2006).

### Local disordered residues

Inspired by the observations of local disorder/order in small segments in a protein, we develop here a formalism, where we state that a residue can be in one of two situations: either an (intrinsically) disordered state (D) corresponding to a non-biased mixture of conformations dictated by the Boltzmann distribution with resulting population-averaged chemical shifts, or in a completely ordered state (O) with a fixed structure. Here we use the simplest probabilistic model, a normal distribution, for the chemical shift in a disordered state. The mean can be estimated from the primary structure as described in Tamiola et al. (2010) and the standard deviation can be derived from statistics (see Methods). Conversely, for an ordered residue, the chemical shifts would have much larger deviation from the mean, corresponding to a bias in the Boltzmann distribution of conformations. Outliers from the normal distribution are indicative of residual order, and can be identified and analyzed for each atom specific chemical shift, but when analyzed in combination the evidence is much more reliable. Therefore, we introduce the *Chemical shift Z-score for assessing Order/Disorder* (the *CheZOD score*), *Z*_*IDR*_, derived from the rmsd of all chemical shift residuals within a residue triplet and linear combinations of fractional powers of this rmsd as described in Methods (Equations 1–4). Assuming that the chemical shifts are normally distributed in disordered residues, *Z*_*IDR*_ is standard normal distributed. This is valid independent of the number of backbone chemical shifts available. Hence, we define the distinction between disordered and ordered residues based on outliers from the normal distribution for *Z*_*IDR*_; a residue is said to be *disordered* if the CheZOD score, *Z*_*IDR*_ < 3, and protein is said to have *local disorder* at this position in the sequence. Furthermore, the CheZOD score is not only a binary classifier of order/disorder, but provides a scale for the “degree of disorder,” which can both classify partially formed/fractionally occupied structure (ca. 3 < *Z*_*IDR*_ < 8) and fully formed structures (*Z*_*IDR*_ > 8).

### Disorder profiles of all proteins in the database

The CheZOD score, *Z*_*IDR*_, was calculated for all residues in all the 117 proteins in the database, henceforth the CheZOD database, and the fraction of the disordered residues was calculated for each protein. We observe that no protein has 100% disordered residues according to our definition. However, five proteins have 95% disordered residues or more, among these are αSyn and Hsp12 (discussed above) and also p15(PAF) (bmrbID = 19332) (De Biasio et al., 2014), the CD3e cytosolic domain of Eukaryota Metazoa Homo sapiens T-cells (bmrbID = 18889; Isaksson et al., 2013), and recombinant murine BG21 isoform of Golli myelin basic protein (bmrbID = 7358; Ahmed et al., 2007). Furthermore, the CheZOD database contains 15, 34, and 70 proteins that have >90, 80, and 50% disordered residues, respectively, for which NMR chemical shifts are available, spanning the full range from disordered to ordered.

**Figure 2:**
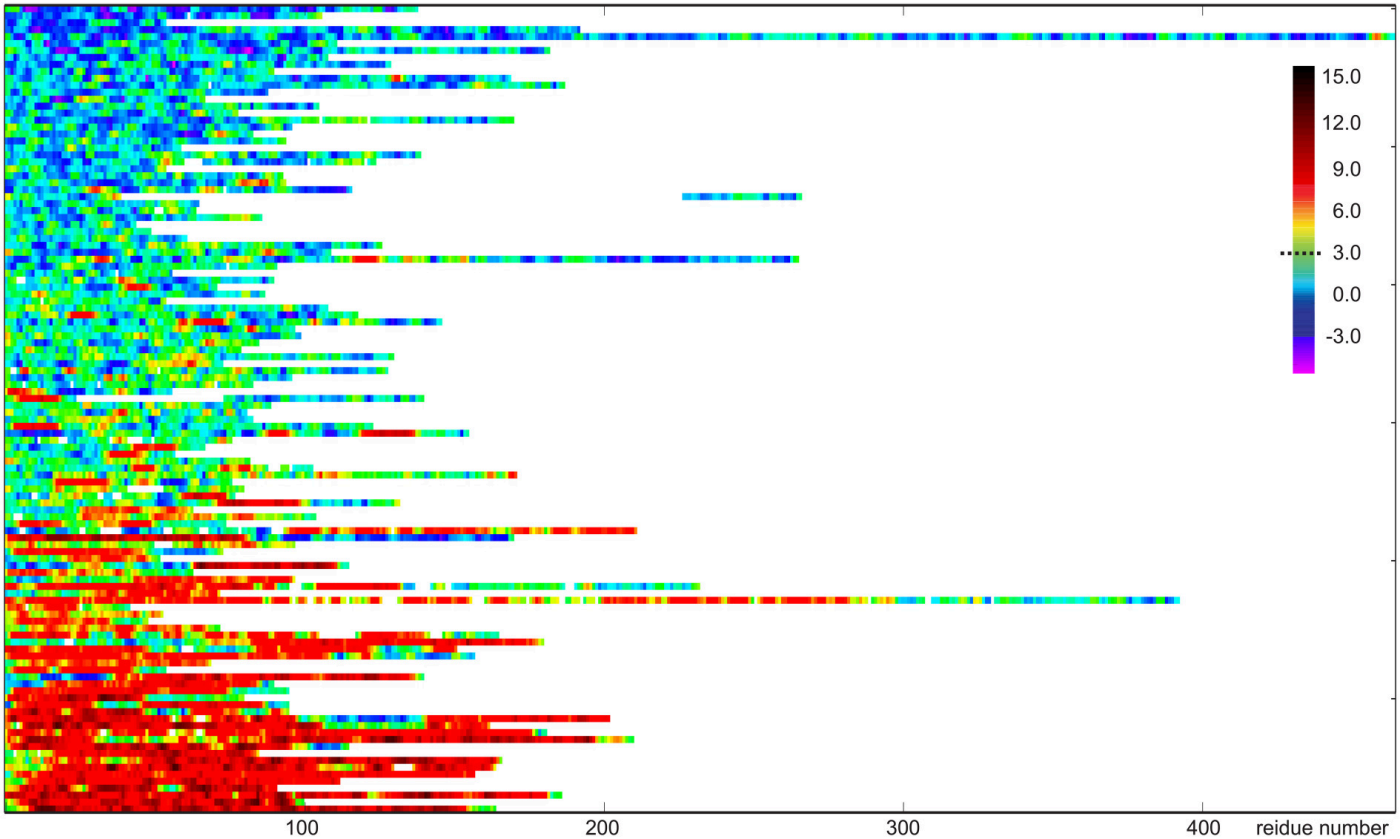
**Visualization of disorder profiles for the 117 proteins sequences in the CheZOD database**. Each row represents a single protein where disordered residues (*Z*_*IDR*_ < 3) are shown in blue, ordered residues (*Z*_*IDR*_ > 3) are shown in red, and residues with average order shown in green/yellow. The proteins are depicted from top to bottom sorted according to the average of Z_*IDR*_ for the protein.

**Figure 3:**
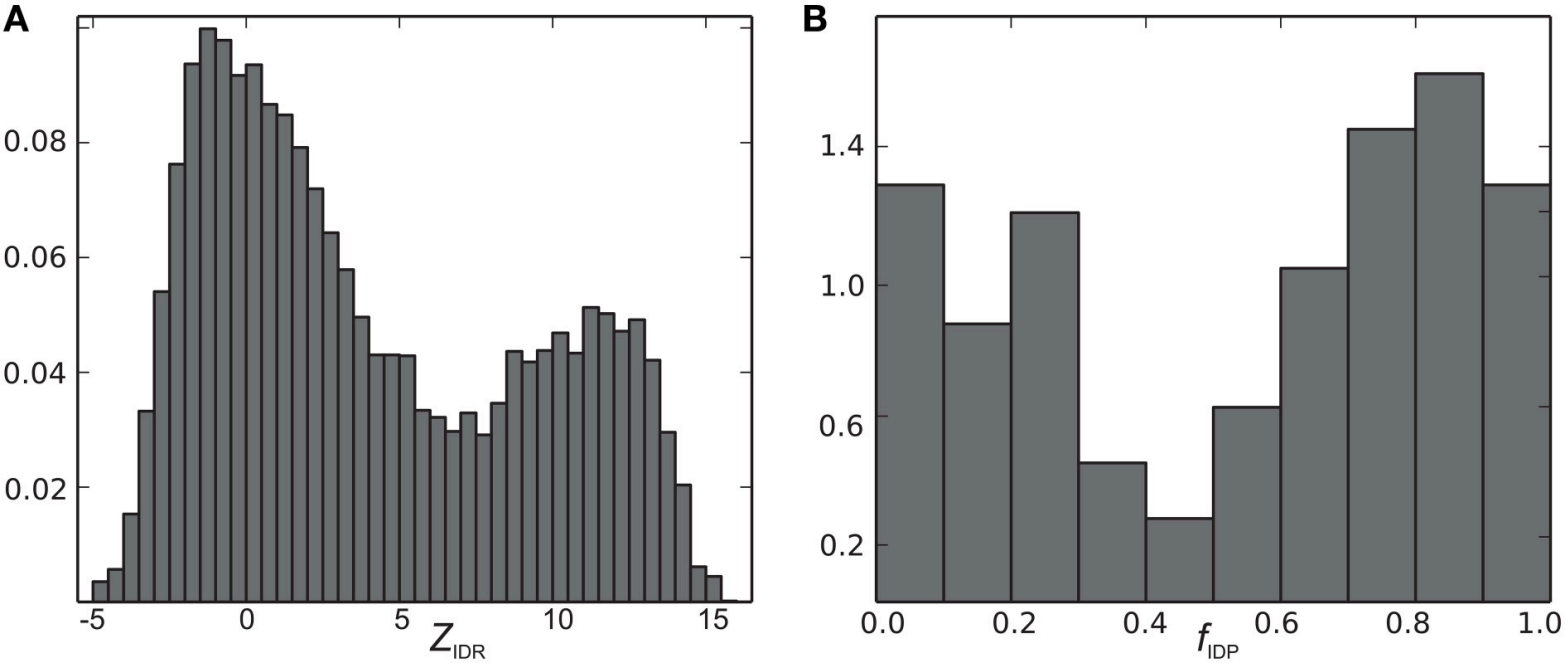
**Histograms of (A) *Z*_*IDR*_ and (B) *f*_IDP_ (normalized to a total area under the curve of 1.0)**.

**Figure 4:**
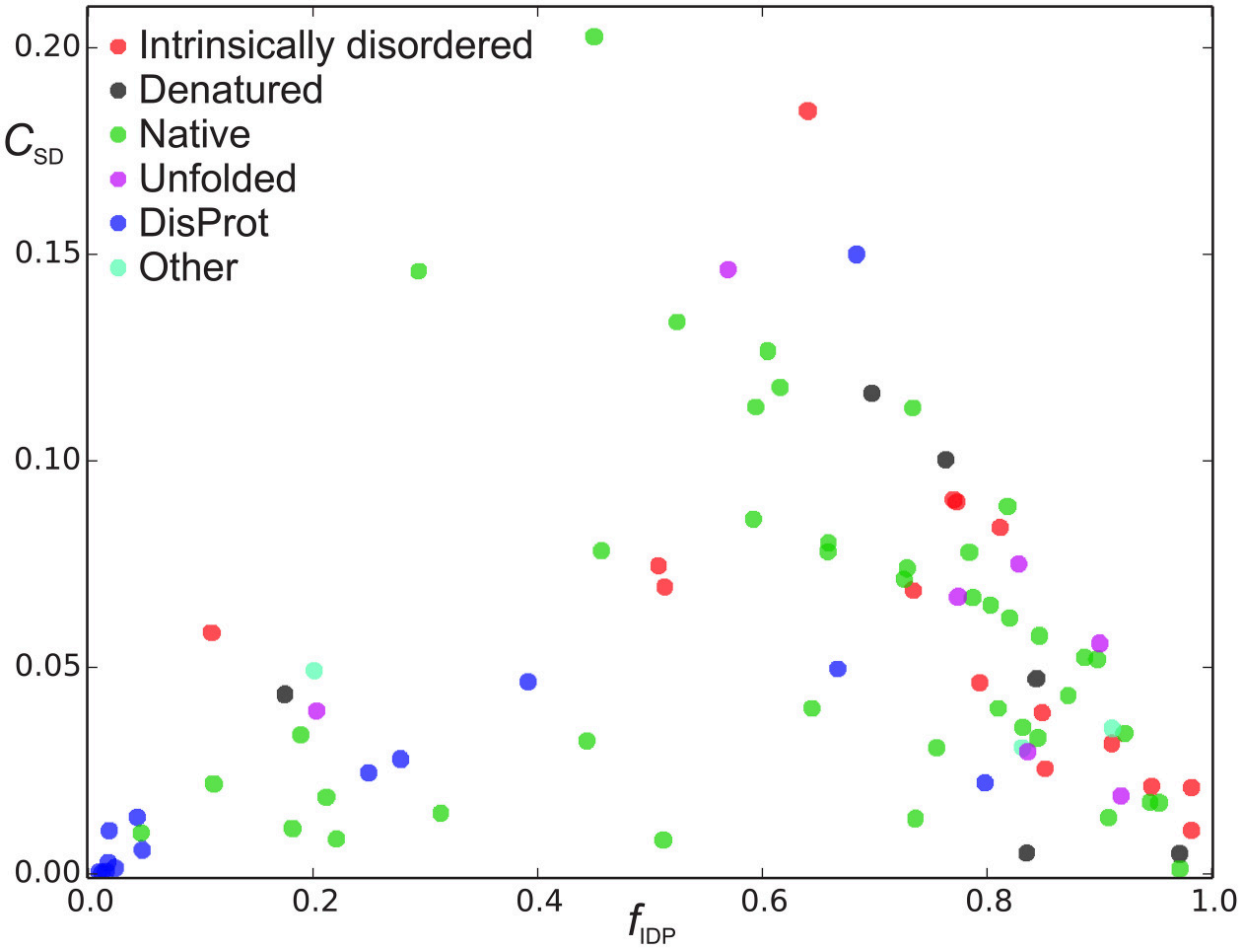
**Sequence disorder complexity, *C*_*SD*_ (Equation 6), as a function of the fraction of disordered residues, *f*_D_**. Each protein is shown with a different color according to the “physical_state” tag provided in the BMRB database. The proteins shown in blue were found in the DisProt database search, where proteins shown in blue had “physical_state” = “native” and cyan points refer to as “unknown” or had missing specification of “physical_state” in the BMRB file. The proteins shown with other colors than blue and gray were found in the BMRB database key word search.

The CheZOD score along the sequence (disorder profile) is visualized in Figure 2 for all proteins in the CheZOD database. It is seen that the database roughly separates into proteins that are mostly disordered, with small segments of order scattered along the sequence, and mostly structured proteins, with small segments of disorder bridging between ordered domains, and in particular at the termini. Conversely, cases with roughly equal amounts of disordered and ordered residues are relatively rare (see also Figures 3A, 4). A second conclusion from our analysis is, that we did not identify proteins where the fluctuations between ordered and disordered residues were completely random. Rather, all proteins have distinct medium-length segments of ordered/disordered residues (with a typical length of 10–30 residues) indicative of short-range correlated behavior, irrespective of the average state of the protein. Careful inspection of Figure 2 reveals that most residues are either fully disordered or fully ordered, whereas fewer are partly ordered (ca. 3 < *Z*_*IDR*_ < 8; see also Figure 3B). Hence, proteins in “the Twilight zone” which are not completely ordered or disordered, both at the local and global level, appear to be under-represented. An example of such a rare protein is the 18.5 kDa isoform of murine myelin basic protein, shown in Figure 1F.

### The sequence disorder complexity of a protein

**Figure 5:**
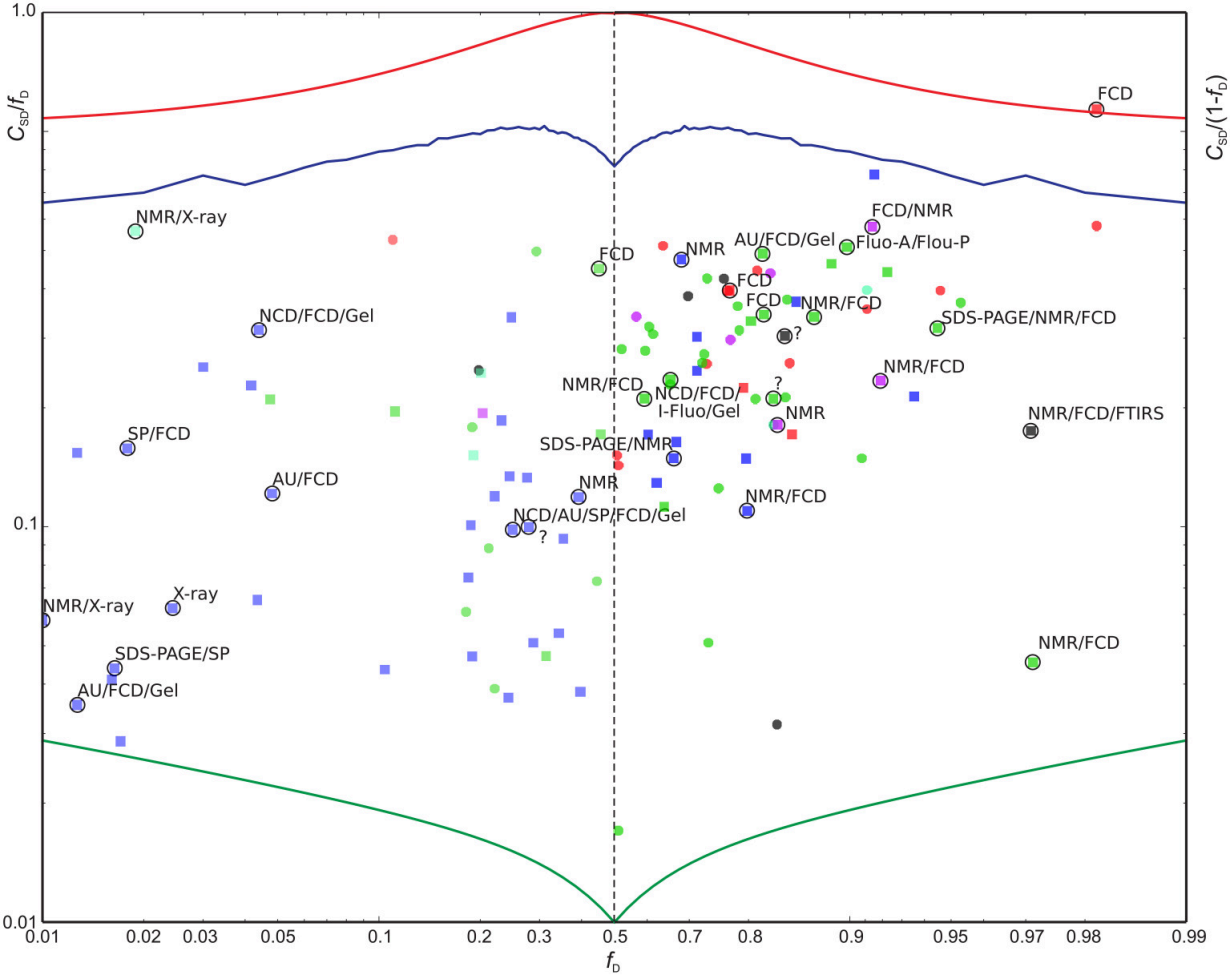
**Scaled sequence disorder complexity *C*_SD_/*f*_D_ as function of *f*_D_ (left) and *C*_SD_/(1−*f*_D_) vs. (1−*f*_D_) (right)**. Extreme values are shown for reference: The minimum possible *C*_SD_ for a protein of length L = 200 (green), the maximum *C*_SD_ (L = 200) (red), and *C*_SD_ for randomly distributed outcomes with a probability *f*_D_ for a disordered residue (blue). Entries highlighted with circles were also found in the DisProt database with confirmed > 90% sequence identity relative to the BRMB database amino acid sequence and with 90% of the aligned residues classified as disordered in DisProt (31 entries). See Figure 4 above for color-coding. The proteins found by searching the DisProt database are shown as squares. The methods used by the DisProt depositors for assessing disorder are provided as annotations near the highlighting circles for the 31 validated entries above. An “?” indicates that no method was given. The following abbreviations were used: AU, Analytical ultracentrifugation; DLS, Dynamic light scattering; EMSA, Electrophoretic Mobility Shift Assay; FCD, Circular dichroism (CD) spectroscopy, far-UV; Fluo-A/Flou-P, Fluorescence polarization/anisotropy; FTIRS, Fourier transform infrared spectroscopy; Gel, Size exclusion/gel filtration chromatography; I-Fluo, Fluorescence, intrinsic; SAXS, Small-angle X-ray scattering (SAXS); SDS-PAGE, Polyacrylamide gel electrophoresis in sodium dodecyl sulfate; SP, Sensitivity to proteolysis.

We now define a measure of the extent of alternations between ordered and disordered segments, the *sequence disorder complexity*, *C*_SD_, which is defined as the Shannon entropy of *Z*_IDR_ < 3 (along the sequence), scaled by the length of the sequence (see Equation 6). *C*_SD_ is 0 for a 100% disordered or ordered protein, and largest for a protein with many alternations between ordered/disordered segments (showing the protein with the maximum sequence disorder complexity in the CheZOD database in Figure 1F). In Figure 4 the *sequence disorder complexity*, *C*_SD_, is shown as a function of the fraction of disordered residues, *f*_D_. For comparison, the relative complexities *C*_SD_/*f*_D_ and *C*_SD_/(1−*f*_D_), which have a more even variation, are shown in Figure 5. It is seen that (i) the distribution of order/disorder along the sequence is not completely random (i.e., the complexity is always much less than the maximum value for *C*_SD_) (ii) The distribution is also not close to minimum complexity, which would correspond to two separate regions of order/disorder. (iii) The relative complexities *C*_SD_/*f*_D_ and *C*_SD_/(1−*f*_D_), as viewed in Figure 5, are asymmetric around *f*_D_ = 0.5. It is seen that mostly disordered proteins (*f*_D_ > 0.5) are more complex compared to their mostly structured counterparts, i.e., they appear to have a relative larger number of scattered smaller segments with local order. However, this apparent asymmetry is mostly due to the specific definition of complexity; i.e., if we choose a different cut-off for a unstructured residue, say *Z*_*IDR*_ > 6 (rather than *Z*_*IDR*_ < 3, Equation 5), the corresponding correlation becomes more symmetric (data not shown) and barely reflects that structured regions are formed internally in the sequence, whereas disordered regions are more typically formed at the termini of the sequence.

### Data extraction and fraction of disordered residues: BMRB vs. disprot

In Figures 4, 5 there are some trends visible in the fraction of disordered residues, *f*_D_, related to the procedure for data extraction and the physical_state tag. In particular, the proteins that have physical_state = “intrinsically disordered” are indeed mostly disordered (*f*_*D*_ ≥ 0.5) in 15 cases, except one. Likewise, proteins corresponding to physical state tags “denatured” and “unfolded” describe only 1 of 6 and 1 of 7 proteins, respectively, that are mostly ordered. This is in some contrast to the group of proteins found from text searches of “disordered” or “unstructured” (see Methods), which were also labeled as “native” (green points in Figures 4, 5). For this group, 10 out of 42 are actually mostly ordered, yet all except one of these proteins still have some degree of disorder with *f*_D_ > 0.1. This bias was even more pronounced when considering the group of proteins identified by searching the DisProt database for corresponding entries in the BMRB database (see Methods). The proteins in this group were labeled with a “native” physical state (blue points in Figures 4, 5) or had no label (2 cases, cyan). The search in the DisProt database also resulted in re-identification of some of the proteins already present in the database from the BMRB physical state tag and text search (shown as colored squares in Figure 5, and not included in the counts below).

Since the DisProt database contains sequences with various contents of disorder, we contend that a protein from DisProt/BMRB is *validated* as disordered if at least 90% of the residues (aligned between the BMRB and DisProt entries) were defined as disordered in the DisProt database. Only the validated Disprot entries are shown in Figure 4 (together with all the BMRB entries), whereas all DisProt entries are shown in Figure 5 highlighting the validated ones by circles. It is seen in Figure 4 that the validated group of DisProt entries contained 11 of 14 mostly ordered proteins, and seven of these were structured proteins, with *f*_D_ < 0.1. It appears that the classification “*mostly disordered*” in DisProt does not assert that a protein, under the conditions corresponding to the BRMB entry, will be disordered, when judged by chemical shift dispersion. However, if the validated entries from DisProt—which correspond to entries already found by the BMRB physical state searches (Figure 5, square points surrounded by circles)—are included in this analysis, the percentage increases, as now 18 of 31 proteins are mostly disordered. Next, we inspected the methods that were used for the disorder classification for the 31 validated DisProt entries (as provided in the DisProt database). A significant correlation is revealed regarding the use of NMR, which was only used in two of the eight cases with almost completely structured proteins, in 3/12 cases for mostly structured, and in 11/19 cases for the mostly disordered proteins (see annotations in Figure 5) suggesting that NMR is likely one of the most accurate methods for assessing disorder in proteins.

The above observations together show that the physical_state tag corresponds well to the content of structure in the protein, and, in particular, using this tag to search for intrinsically disordered proteins is a reliable procedure for identifying IDPs. Although the tags “intrinsically disordered,” “unfolded,” and “denatured” all consistently yield disordered proteins, one could suspect that in the latter two cases the protein might be in a somewhat non-native state, biased by conditions that could induce unfolding/denaturation of the protein (although it was specifically tried to avoid this, by excluding entries where it was specified that denaturants or co-factors were added, see Methods). This impression is supported by the fact that all six “denatured” proteins in the CheZOD database, and four of seven of the “unfolded” proteins, also have a structure available in the PDB database (as defined by the cross-ref PDB match in the BMRB entry), indicating that a folded version of the protein exists under some condition. For comparison, only 2 of 15 proteins with “intrinsically disordered” physical state have a folded structure in the PDB. An alternative procedure to identify “truly disordered” proteins, as demonstrated here, would be to search in the BMRB title for the words “disordered” or “unstructured” and analyze the proteins with a “native” physical state (green points in Figures 4, 5). In this case, only 15 out of 42 had a folded structure available in the PDB database. In general, there is a close relationship between the fraction of disordered residues and the presence of a folded structure in the PDB; 26 of 70 of the mostly disordered proteins have a determined structure, whereas a vast excess of 44 of 47 from the mostly ordered proteins have a structure present in the PDB.

## Discussion

We have constructed here the CheZOD database that contains detailed information about the extent of disorder in 117 carefully curated protein data entries. Following our bottom-up approach to include all available data for potentially disordered proteins in the BMRB database, it was intended that the CheZOD database should be representative for the full range of intrinsically disordered proteins. In the quest for this we used both tag searches in BMRB for the physical state, as well as searches to find chemical shifts for entries in the DisProt database. The CheZOD database is relatively small compared to other databases with 117 proteins, but one can imagine that the CheZOD database could be expanded even further in the future by including searches for entries in other databases such as MobiDB (Potenza et al., 2014), IDEAL (Fukuchi et al., 2014), and D^2^P^2^ (Oates et al., 2013). We also expect further entries with chemical shifts to be available for inclusion in the CheZOD database in the near future following the recent growing focus on IDP research and advances in NMR analysis of IDPs. Despite the efforts made here, we do not claim here that our database is complete in the sense that it would cover all possible classes of proteins or types of disorder. We also concede that our database is slightly “biased” as a whole, in the sense that all entries correspond to proteins amenable to NMR spectroscopy, such as soluble proteins with small/medium size at relatively high concentrations. Notwithstanding these subtle objections, we still argue that the CheZOD database is *representative* for protein disorder. All entries in the CheZOD database is summarized in Table S1, and the full database including all the CheZOD Z-scores and backbone chemical shifts for each residues is available from www.protein-nmr.org.

We have used here our method for chemical shift referencing based on recalibration of the distribution of chemical shift for the disordered residues, and the random neighbor corrected chemical shifts of Tamiola et al. (2010). We now compare with the LACS method for re-referencing chemical shifts (Wang et al., 2005), Kjaergaard et al. random coil chemical shifts (Kjaergaard and Poulsen, 2011), and the RCI method for estimating local dynamics based on the chemical shifts (Berjanskii and Wishart, 2005).

Our method for re-referencing the chemical shifts identifies the disordered residues and calculates the average secondary chemical shifts, Δ_ave_, for these residues. In theory, Δ_ave_ must be very close to zero, but it will deviate from zero for several possible reasons: (i) incorrect referencing by the authors of the entry, (ii) influence by sample conditions such as isotope effects, solvent, buffer, temperature, and pH, (iii) systematic bias in the estimation of neighbor corrected random coil shifts, (iv) small “true deviation” due to residual structure bias. Our method aims at addressing (i–iii) with a phenomenological offset correction by subtracting the average value, Δ_ave_, from the chemical shifts. While some of the effects might be rather small, they could still have a dramatic impact on the interpretation of disorder due to the small variation in secondary shifts for IDPs. Unfortunately, it is of course not possible to separate effects of (i–iii) from “true deviation” which could mask structural signatures and thereby the disordered residues would appear slightly more disordered. We note, however, that this re-referencing does not lead to a larger number of residues being classified as disordered but only effects the amplitude of the disorder, merely leading to a slight skewing of the Z-score scale in the low-value end of the scale. Following the rationale that the effect of (i–iii) would often be much larger than the “true deviation” correction, this is why we applied the offset correction exclusively in cases where it was significantly different from zero as judged by Akaikes information criterion.

Analysis of all the applied offset corrections for the full CheZOD database by each atom type reveals that the offset correction was used in 20.6% of the chemical shift sets when analyzing the individual atom types separately (see Figure S1 in the Supplementary Material). In most cases the offset corrections were small (< 0.5 ppm for ^13^C and ^15^N and < 0.15 ppm for ^1^H) but for a few entries, a large offset correction (>2 ppm) was needed for the carbon atom types. In these cases with large corrections, the offset for the different carbon atom types were correlated, underscoring the credibility of our method for offset corrections although these chemical shifts are sometimes assigned from different spectra. Some systematic trends in the sign of the offset correction can be learned, in particular the Hα and H_N_ correction were often negative and positive, respectively, by ca. 0.1 ppm (discussed in more detail below). Furthermore, it is seen that offsets were more frequently used for more disordered proteins, which was to be expected, since the offset is calculated using only the disordered residues and hence, a deviation from the expected mean would be more statistically significant according to Akaikes information criterion, which is proportional to the number of data points.

In order to provide independent validation of our method, we compared our offset corrections to offset corrections estimated by LACS (Wang et al., 2005), and found that the two methods agree exceedingly well (see Figure S2 in the Supplementary Material), with the exception of the Hα shift, for which there was a small systematic deviation, which is, however, accounted for when using our phenomenological offset correction method. These Hα offsets predicted by our method were ca. −0.1 ppm on average whereas the offsets for the same entries were ca. +0.1 ppm although still apparently linearly related. This observation suggests small differences in the reference values used for Hα random coil chemical shifts for the two methods. There were some notable differences between our method and LACS that implicate that it cannot be applied in all cases; in particular LACS requires assigned Cα and Cβ chemical shifts and only provides offset correction estimates for Cα, Cβ, Hα, and C′.

To address the possibility that observed chemical shifts could be affected by sample conditions, we compared our secondary chemical shifts and Z-scores with the corresponding results obtained from derivations using the random coil chemical shifts from Kjaergaard et al. (Kjaergaard and Poulsen, 2011), which includes an estimate of the effect due to variations in temperature and pH. Comparing the results for our seven test case entries in Figure 1, there were only some small differences for the disordered residues where the secondary chemical shifts were small compared to the difference between the random coil shift estimates, and elsewhere largely very similar results for the two methods (see Figure S3 in the Supplementary Material). This observation suggests that our method already includes the effects on chemical shifts from pH and temperature implicitly. However, since it was not possible to account for all effects of the sample conditions on the chemical shifts or for cooperative/non-linear effects of the different parameters, we still argue that our phenomenological correction is the most appropriate.

NMR is very sensitive to ensemble averaging of local conformations, which is measured very accurately from the chemical shifts. Therefore, chemical-shift based methods for assessing order/disorder in proteins are both position-specific, and also provide a scale for the extent of disorder/order. These properties contrast with other biophysical techniques such as circular dichroism (CD), and infra-red (IR) spectroscopies, aimed at estimating the content of secondary structure, or techniques for estimating the protein size, density, hydrodynamic drag, or diffusion properties, using for example, size exclusion chromatography, ultracentrifugation, SAXS, or limited proteolysis (Vucetic et al., 2005; Tompa, 2009; Uversky and Longi, 2010). Missing density in X-ray crystallography data may also be indicative of local disorder (though it doesn't provide a scale for the disorder), but X-ray diffraction cannot be applied to proteins that do not contain any structured elements. Hence, with the chemical shift analysis proposed here, it is possible to “zoom-in” to look for finer details, and get a more detailed picture of the diversity in disorder.

Some other methods also provide residue based estimates for the local dynamics including δ2D (Camilloni et al., 2012) and the Random Coil Index (RCI) method (Berjanskii and Wishart, 2005). We have compared our Z-score for our seven test case proteins to the RCI estimates of the S^2^ local order parameter in Figure S3. There was qualitatively agreement between the RCI estimated S^2^ and our *Z*-score, i.e., low order for the disordered residue and high order (S^2^ close to 1) for the ordered residues. However, there were still some more subtle differences between the two scores. Firstly, the largest differences were for the disordered residues where the secondary chemical shifts were small compared to the difference between the random coil shift estimates. This can be understood, since RCI uses a floor value for the absolute chemical shifts meaning that very small deviations from the chemical shifts are not captured and hence also very disordered residues are not distinguished. Secondly, the full range of orders appears to be less well described by the RCI estimates yielding only small differences for fully and partly formed structure (see e.g., Figure S3d). Thirdly, the RCI method gives a smoother trajectory along the sequence, which is because it uses both a three-point averaging of the absolute secondary chemical shifts as well as for the actual RCI index values. Finally, we note that whereas the RCI method is heuristic, based on a weighted sum of the absolute value of secondary chemical shifts for all atom types, our method is statistical, based on the chi square distribution of squared secondary chemical shifts, and due to this formulation it provides adequate estimates for the local dynamics also in cases were only a subset of the chemical shifts are available such as only proton or carbon chemical shifts.

Inspection of our CheZOD database of IDPs reveals that the proteins span a broad range of fractions of disorder and extent of disorder. Proteins are seen with both many fluctuations between segments of order/disorder, characterized by a high sequence disorder entropy, and with separation into larger completely ordered/disordered domains. This great diversity in disorder reflects the broad class of IDPs known to form various functions or interaction with different targets and malleability at different conditions.

Proteins that are either almost completely disordered, or completely structured, are most abundant in the CheZOD database, whereas cases with roughly equally mixed states are rare. This observation likely reflects the cooperative behavior in order/disorder transitions, where formation of ordered segments promotes the formation of other ordered segments and vice versa. Following this line of thought, proteins in the “Twilight Zone” might reflect folding intermediates on a transition path from unfolded to folded. The seemingly cooperative nature of disorder also provides a clue to why it has been so difficult to construct predictive models for disorder from local amino acid composition alone. It is at current difficult to address whether the formation of medium length segments of alternating order/disorder are due to a cooperative transition together with nearest neighbors or due to properties of the amino acids in the segment.

The analysis of the CheZOD database revealed that entries found in the DisProt database were often mostly structured. The higher degree of structure for these protein entries could be due to differences between the sample conditions during analysis corresponding to the BMRB and DisProt entries. For example, the NMR analysis corresponding to the BMRB entry could be performed under conditions that favor structure determination. Alternatively, one could speculate that the identification of disordered regions, leading to the inclusion in the DisProt database, could be based on methods that are less strict compared to NMR or more “coarse-grained,” in the sense that they only provide a classification for the full protein and not at a local level. In support of this, we found that DisProt entries, which used NMR to assess disorder, were also more often confirmed to be disordered in our analysis. Hence, a more critical assessment of disorder based on a more reliable and uniform criterion, such as can be derived with NMR, is recommended.

### Implications for the bioinformatics analysis of protein disorder

The last years have seen a tremendous increase in the number of bioinformatics tools that try to predict protein (dis)order (Schlessinger et al., 2009; Dosztányi et al., 2010) and several prediction methods participated in recent rounds of the Critical Assessment of Structure Predictions (CASP; Noivirt-Brik et al., 2009; Monastyrskyy et al., 2011). Unfortunately, current datasets of experimentally classified ordered and disordered regions (Sickmeier et al., 2007) contain many misclassified segments: In X-ray crystal structures regions may appear ordered due to binding partners or crystal packing forces, and could be disordered in isolation. In disorder databases segments may even be more prone to misclassification, since many longer disordered regions are characterized by semi-quantitative experiments that lack position specific information. This suspicion of misclassification was confirmed in our analysis of the validated entries from the DisProt database where several entries where almost completely structured in the CheZOD database. Furthermore, the order/disorder status is also sensitive to environmental conditions, and this fact is not considered. The lack of sufficiently reliable datasets and the noise in the assignment of order and disorder represent a serious limitation in developing accurate prediction methods for protein disorder (Dosztányi et al., 2010). The final, serious shortcoming, of current prediction methods is their inaccuracy when going down to shorter stretches (preliminary analysis). This study reveals a preponderance of proteins with mixed ordered and disordered segments and high sequence disorder complexity—typically, for proteins with mixed order, *C*_*SD*_ ≈ 0.1 corresponding to an average segment length of ca. 10 residues.

The CheZOD database presented here was carefully manually curated to exclude any entries with biasing conditions and strictly contains proteins under native conditions. Here the analysis and processing of chemical shifts provides a unique experimentally validated local and quantitative measure of order/disorder. Furthermore, our database covers a broad range of proteins ranging from completely ordered to almost completely structureless. Since predictions in CASP9 were no better than those in CASP8 (Monastyrskyy et al., 2011), and only a small improvement was noted for CASP10 (Monastyrskyy et al., 2014), we hope that our curated CheZOD database can help form the basis for the development of even more accurate and sophisticated predictive models of order/disorder. This will enable us to ask more detailed questions and provide answers to complex biologically relevant problems related to intrinsically disordered proteins.

## Conclusions

We have used a systematic analysis of NMR chemical shifts to build a database of experimentally validated disordered proteins, the CheZOD database. In contrast to other methods for order classification, our procedure provides a reliable position-specific quantitative measure of order/disorder through our *Chemical shift Z-score for assessing Order/Disorder* (the *CheZOD score*). Examples were observed of both maximum CheZOD score in completely ordered segments, intermediate values in loops and fractionally populated structure, and small values in completely disordered regions. Careful inspection and systematic analysis of the entries in the CheZOD database revealed interesting trends and variations. In particular, it was discussed here how proteins can be completely disordered, partially disordered, or only disordered in a small segment. Some proteins can indeed be classified as *super unfolded*, like human α-synuclein, indicating that this protein is not an archetypal IDP. Through the introduction of the sequence disorder complexity we found diverse patterns of disorder, e.g., that proteins can be segregated into two distinct parts of an ordered and a disordered domain, but also be composed of smaller segments varying alternatingly between order and disorder. A typical segment length of ordered/disordered residues was estimated to be ca. 10 residues. We foresee that further systematic analysis of our CheZOD database will contribute to a more detailed understanding of the relationship between primary sequence and disorder/structure and function.

## Author contributions

Both authors JTN and FM contributed to designing and developing the project and writing the paper. JTN performed the data collection, processing, and analysis by building the database of (intrinsically disordered) proteins with assigned chemical shifts, calculating the chemical shift Z-score indicative of disorder, and analyzing the trends in the database.

### Conflict of interest statement

The authors declare that the research was conducted in the absence of any commercial or financial relationships that could be construed as a potential conflict of interest. The reviewer WV and Handling Editor declared their shared affiliation, and the Handling Editor states that the process nevertheless met the standards of a fair and objective review.
