# Supplementary material for: There is Diversity in Disorder—“In all Chaos there is a Cosmos, in all Disorder a Secret Order”
Source: Front Mol Biosci. 2016 Feb 11;3:4. doi: 10.3389/fmolb.2016.00004 (PMC4749933; doi:10.3389/fmolb.2016.00004)
Supplement: Supplementary file 1 [file DataSheet1.DOCX]

# There is Diversity in Disorder – “In all chaos there is a cosmos, in all disorder a secret order”^#^

Jakob Toudahl Nielsen* and Frans A.A. Mulder

Department of Chemistry and Interdisciplinary Nanoscience Center (iNANO), University of Aarhus, Gustav Wieds Vej 14, DK-8000 Aarhus C, Denmark

## Supporting information

**Contents**:

Figures S1, S2 and S3

Table S1

**Fig. S1: All used chemical shift offset corrections**. The recalibrated offset correction used in this study as a function of protein rank according to average Z-score (as in Figure 2) using blue, red, black, green, cyan, magenta and yellow dots for C´, Cα, Cβ, Hα, H_N_, N and Hβ, respectively (as in Fig. 1). The corrected chemical shifts were obtained by adding these offsets to the observed chemical shifts.

**Fig. S2**: **Comparison of CheZOD and LACS offset corrections**. The offset when used in the present study as a function of the LACS offset estimation.

**Fig. S3**: **Comparison of applied random coil shift and RCI S^2^ predictions**. The CheZOD Z-score is shown with a black line, and a similar Z-score, calculated using secondary chemical shift obtained by using the method of Kjaergaard et al. for neighbor, pH and temperature corrected random coil chemical shifts, is shown with a red line. The order parameter, S^2^ estimated by the random coil index method by Wishart et al. is shown with a blue line. To ease comparison 15.0 * S^2^ - 5.0 is plotted.

**Table S1: The CheZOD database**.

| bmrID | Physical state | Shifts (number) | *f*_D_ | c_SD_ | pH | Temp (K) | Protein name |
| --- | --- | --- | --- | --- | --- | --- | --- |
| 19332 | Intrinsic^a^ | 619 | 0.9815 | 0.01066 | 6.6 | 298.0 | p15(PAF) |
| 18889 | intrinsic | 241 | 0.9815 | 0.02101 | 6.8 | 298.0 | CD3e cytosolyc domain |
| 17483 | native | 673 | 0.9714 | 0.00130 | 7.0 | 298.0 | Small heat shock protein (Hsp12) |
| 6968 | denatured | 924 | 0.9710 | 0.00507 | 6.5 | 285.5 | alpha-synuclein |
| 7358 | native | 1043 | 0.9531 | 0.01727 | 6.8 | 280.0 | Golli myelin basic protein isoform BG21 |
| 19135 | intrinsic | 2682 | 0.9462 | 0.02129 | 6.9 | 300.1 | MAP2c |
| 16876 | native | 1032 | 0.9451 | 0.01744 | 6.5 | 288.0 | Dehydrin ERD14 |
| 15336 | native | 526 | 0.9355 | 0.01380 | 6.8 | 283.0 | Nonphosphorylated CFTR Regulatory Region |
| 17290 | native | 830 | 0.9225 | 0.03410 | 6.0 | 298.0 | Cytoplasmic domain of humanneuroligin-3 |
| 15409 | unfolded | 529 | 0.9189 | 0.01897 | 6.7 | 288.0 | Cytoplasmic domain of the T cell receptor zeta chain |
| 15441 | native | 217 | 0.9153 | 0.06579 | 6.5 | 293.0 | Dynein intermediate chain IC74 (res 84-143) |
| 7279 | unfolded | 514 | 0.9143 | 0.04912 | 6.0 | 298.0 | Human non-chromitin architectural transcription factor HMGA1 |
| 7292 | partial^b^ | 772 | 0.9112 | 0.03524 | 4.0 | 300.0 | Internal interaction site of ALP |
| 19357 | intrinsic | 426 | 0.9111 | 0.03158 | 6.8 | 273.0 | Met66 prodomain region of BDNF |
| 18927 | native | 297 | 0.9077 | 0.01375 | 6.0 | 298.0 | V5 domain from Protein Kinase C alpha |
| 19650 | native | 233 | 0.8980 | 0.05199 | 7.0 | 283.0 | Cytosolic domain of CD79b |
| 15506 | native | 321 | 0.8864 | 0.05256 | 6.8 | 288.0 | Translocation domain of colicin N |
| 15274 | native | 361 | 0.8723 | 0.04330 | 6.0 | 298.0 | Arf-binding domain of Hdm2 |
| 17760 | native | 378 | 0.8556 | 0.05355 | 7.4 | 293.0 | p53 N-terminal transactivation domain |
| 11454 | intrinsic | 586 | 0.8512 | 0.02544 | 7.3 | 277.0 | QB domain of Sp1 |
| 18198 | intrinsic | 325 | 0.8488 | 0.03918 | 6.6 | 298.0 | PP1 Binding Domain of NIPP1 |
| 18631 | native | 368 | 0.8462 | 0.05764 | 7.2 | 298.0 | Sulfydryl Oxidase of ALR (N-term) |
| 17836 | native | 550 | 0.8448 | 0.03293 | 7.5 | 298.0 | apo-IscU |
| 4286 | denatured | 353 | 0.8438 | 0.04739 | 4.5 | 288.0 | Vesicular SNARE Snc1. |
| 15397 | unfolded | 304 | 0.8358 | 0.02970 | 6.7 | 304.1 | Nuclear hormone receptor coactivator ACTR (res 1018-1088) |
| 4287 | denatured | 294 | 0.8354 | 0.00521 | 4.5 | 288.0 | Target Membrane SNARE Sso1. |
| 16296 | native | 518 | 0.8313 | 0.03561 | 7.0 | 298.0 | Homo sapiens FCP1 (res 877-961) |
| 19796 | molten | 284 | 0.8310 | 0.03058 | 7.4 | 273.0 | Hinge region of human Col7 |
| 15563 | unfolded | 429 | 0.8280 | 0.07515 | 4.5 | 293.0 | Human SRC (res 1-85) |
| 18867 | native | 295 | 0.8197 | 0.06206 | 6.7 | 298.0 | CD79a cytosolic domain |
| 17048 | native | 273 | 0.8182 | 0.08907 | 7.0 | 300.0 | AP180 fragment (AP180 M5) |
| 18248 | intrinsic | 227 | 0.8113 | 0.08380 | 6.0 | 298.0 | Dually targeting peptide from Thr-tRNA synthetase |
| 19114 | native | 679 | 0.8095 | 0.04008 | 6.0 | 283.0 | 4E-BP2 |
| 6078 | native | 273 | 0.8033 | 0.06517 | 5.6 | 293.0 | IA3, an Aspartic Proteinase Inhibitor for Saccharomyces cerevisiae |
| 4272 | native | 590 | 0.7979 | 0.02220 | 6.1 | 298.0 | SynaptobrevinII |
| 4950 | native | 155 | 0.7963 | 0.03030 | 4.6 | 285.0 | Non-myristoylated Nef anchor domain (res 2-57) |
| 19364 | intrinsic | 406 | 0.7931 | 0.04639 | 4.5 | 298.0 | Nupr1 |
| 19171 | native | 534 | 0.7870 | 0.06683 | 6.5 | 298.0 | Transcriptional repressor domain of methylated DNA binding domain protein 1 |
| 15766 | native | 609 | 0.7842 | 0.07798 | 6.1 | 298.0 | Human calpastatin Domain 1 |
| 15136 | unfolded | 471 | 0.7739 | 0.06713 | 7.5 | 283.0 | Endosulfine alpha |
| 18890 | intrinsic | 214 | 0.7727 | 0.08997 | 6.8 | 298.0 | CD3g cytosolic domain |
| 19478 | intrinsic | 281 | 0.7701 | 0.09066 | 6.5 | 278.0 | Human GW-protein TNRC6B motif I |
| 4922 | denatured | 619 | 0.7629 | 0.10037 | 6.0 | 308.0 | apo-plastocyanin |
| 26549 | native | 1353 | 0.7547 | 0.03066 | 6.5 | 298.0 | HCV protein NS5A (res 191-447) |
| 18177 | native | 400 | 0.7363 | 0.01342 | 7.0 | 273.0 | cVIMP-Cys |
| 15180 | native | 517 | 0.7342 | 0.11279 | 6.5 | 298.0 | Spinophilin PP1 binding domain (res417 - 494) |
| 19507 | intrinsic | 535 | 0.7339 | 0.06859 | 6.5 | 290.0 | Culture Filtrate Antigen protein (CFP10) from Mycobacterium Tuberculosis |
| 15176 | native | 772 | 0.7288 | 0.07403 | 5.5 | 298.0 | Darpp-32 (res 1-118) |
| 11526 | native | 974 | 0.7260 | 0.07123 | 6.5 | 303.0 | Yeast Ump1 |
| 6431 | native | 791 | 0.7154 | 0.07070 | 6.5 | 278.0 | II-III loop region of the skeletal dyhydropyridine receptor |
| 5313 | native | 862 | 0.7154 | 0.08619 | 5.8 | 280.0 | Rat Gap Junction 43 kDa carboxyl terminal domain |
| 19672 | denatured | 311 | 0.6970 | 0.11623 | 6.0 | 298.0 | PTB Domain of Dok1 |
| 16505 | native | 312 | 0.6835 | 0.14995 | 5.0 | 298.0 | Syrian hamster prion protein (res 57-91) |
| 17205 | native | 325 | 0.6716 | 0.05380 | 7.0 | 298.0 | Cox17 from Yeast |
| 15123 | native | 331 | 0.6667 | 0.04961 | 6.0 | 298.0 | SeV Ntail (res 443-501) |
| 15430 | native | 529 | 0.6588 | 0.08029 | 4.0 | 298.0 | Gamma subunit of phosphodiesterase |
| 15179 | native | 1026 | 0.6581 | 0.07808 | 6.5 | 298.0 | Phosphatase 1 Inhibitor-2 |
| 25118 | native | 443 | 0.6438 | 0.04008 | 6.2 | 313.0 | Human cardiac troponin I |
| 18446 | intrinsic | 360 | 0.6404 | 0.18466 | 7.5 | 298.0 | Crystallin from Hahella chejuensis |
| 6846 | native | 366 | 0.6250 | 0.04850 | 5.0 | 298.0 | Nop10p from Saccharomyces cerevisiae |
| 16450 | native | 740 | 0.6160 | 0.11787 | 6.0 | 300.0 | YSK2 |
| 25327 | native | 455 | 0.6049 | 0.12656 | 7.0 | 298.0 | Bd0108 from Bdellovibrio bacteriovorus |
| 6112 | native | 227 | 0.6024 | 0.06812 | 6.5 | 298.0 | p27-KID |
| 15719 | native | 493 | 0.5938 | 0.11302 | 7.0 | 278.0 | Human h1 calponin (res 131-228) |
| 15141 | native | 379 | 0.5918 | 0.08596 | 5.0 | 298.0 | Thyroid Cancer 1 protein |
| 5076 | unfolded | 156 | 0.5692 | 0.14639 | 6.7 | 303.0 | Cold Shock Domain of the human YB-1 protein |
| n.a. | native | 530 | 0.5244 | 0.13359 | 5.0 | 278.0 | Heavy metal binding domain of Lp CopA |
| 19485 | intrinsic | 478 | 0.5132 | 0.06956 | 5.0 | 298.0 | Small VCP/p97-interacting protein (Human) |
| 16912 | native | 1170 | 0.5118 | 0.00833 | 6.6 | 301.0 | Delta subunit of RNA polymerase from Bacillus subtilis |
| 18578 | intrinsic | 855 | 0.5076 | 0.07470 | 5.8 | 310.0 | Carboxyl Terminal Domain of the Connexin45 Isoform |
| 17926 | native | 342 | 0.4571 | 0.07816 | 7.0 | 293.0 | C-term of Arab Thaliana CP12-2 |
| 15131 | native | 848 | 0.4503 | 0.20257 | 6.5 | 277.0 | Murine myelin basic protein |
| 17325 | native | 775 | 0.4435 | 0.03229 | 6.0 | 303.0 | Engrailed 2 (res 1-117) |
| 11019 | native | 170 | 0.3968 | 0.01520 | 7.2 | 298.0 | Cox17 |
| 6212 | native | 424 | 0.3919 | 0.04663 | 6.5 | 300.0 | Subunit F6 from the peripheral stalk region of ATP synthase |
| 5736 | native | 894 | 0.3532 | 0.03288 | 7.0 | 298.0 | ICln ion channel cloned from epithelial cells |
| 6498 | native | 281 | 0.3425 | 0.01842 | 7.0 | 303.0 | SIP (1-77) |
| 15672 | native | 472 | 0.3138 | 0.01480 | 5.5 | 308.0 | MMLV p12-CA(NTD) |
| 15768 | native | 253 | 0.2941 | 0.14589 | 6.6 | 278.0 | N-terminal half of hepatitis core protein |
| 4716 | native | 859 | 0.2881 | 0.01466 | 7.0 | 298.0 | C-terminal Recombinant Fragment of Auxilin Including the J-domain |
| 16160 | native | 356 | 0.2784 | 0.02782 | 6.8 | 283.0 | Myosin Phosphatase Trageting Subunit 1 (res 1-98) |
| 25093 | native | 517 | 0.2750 | 0.03667 | 6.5 | 298.0 | EcMazE |
| 5204 | native | 403 | 0.2500 | 0.02458 | 6.3 | 293.4 | Calreticulin P-domain fragment (res 189-261) |
| 5545 | native | 276 | 0.2475 | 0.08381 | 4.5 | 298.0 | Insulin-like growth factor binding protein-6 (res 161-240) |
| 15711 | native | 663 | 0.2449 | 0.03291 | 6.3 | 298.0 | Monomeric apo-SOD1 |
| 4307 | native | 803 | 0.2429 | 0.00899 | 5.2 | 298.0 | SHa rPrP (res 90-231) |
| 6521 | native | 144 | 0.2308 | 0.04287 | 6.0 | 293.0 | p53 tetramerization domain |
| 4034 | native | 452 | 0.2211 | 0.00861 | 6.6 | 300.0 | Monomer-Binding Nuclear Orphan Receptor, Human ERR2 (res 97-194) |
| 16670 | native | 538 | 0.2211 | 0.02641 | 6.5 | 293.0 | Apoptosis- and inflammation-related NALP1 pyrin domain |
| 19224 | native | 1121 | 0.2120 | 0.01867 | 6.8 | 298.0 | Phosphatase 1B (PTP1B) (res 1-393) |
| 15398 | unfolded | 197 | 0.2037 | 0.03954 | 6.7 | 304.1 | Nuclear coactivator binding domain of CBP |
| 15162 | molten^c^ | 648 | 0.2013 | 0.04929 | 7.2 | 288.0 | Cholera Toxin Enzymatic Domain (1-167) |
| 1128 | unknown^d^ | 177 | 0.1905 | 0.02884 | 4.5 | 295.0 | Recombinant Desulfatohirudi |
| 15086 | native | 649 | 0.1895 | 0.03379 | 4.4 | 298.0 | Hypothetical Protein Cgl2762 from Corynebacterium Glutamicum |
| 4280 | native | 500 | 0.1889 | 0.00889 | 6.0 | 291.0 | Methyl Binding Domain of the Methyl-CpG-binding Protein MeCP2 |
| 6139 | native | 188 | 0.1875 | 0.01895 | 6.0 | 308.0 | Ole e 6 |
| 6974 | native | 592 | 0.1847 | 0.01372 | 6.8 | 293.0 | Human p23 (res 1-160) |
| 15097 | native | 1057 | 0.1818 | 0.01109 | 7.0 | 298.0 | D6-HP of chicken villin |
| 15174 | denatured | 139 | 0.1750 | 0.04351 | 5.9 | 298.0 | [L-Phe44]iota-RXIA |
| 6580 | native | 914 | 0.1118 | 0.02188 | 6.7 | 298.0 | Mouse socs3 |
| 19037 | intrinsic | 513 | 0.1099 | 0.05835 | 7.0 | 298.0 | h-prune (res 354-453) |
| 11388 | native | 751 | 0.1043 | 0.00454 | 7.0 | 298.0 | ARID domain of Jarid1b protein |
| 5956 | native | 464 | 0.0482 | 0.00585 | 6.8 | 308.0 | Max*VL subunit 1 of b-HLH-LZ |
| 19521 | native | 1349 | 0.0476 | 0.01001 | 7.0 | 298.0 | BCL-xL |
| 19482 | native | 599 | 0.0440 | 0.01383 | 7.0 | 300.0 | Regulatory Domain of Tyrosine Hydroxylase |
| 4914 | native | 196 | 0.0435 | 0.00284 | 5.0 | 303.0 | vMIP-II (res 1-71) |
| 4686 | native | 207 | 0.0417 | 0.00948 | 5.0 | 303.0 | Human CC Chemokine I-309 |
| 15736 | native | 957 | 0.0301 | 0.00763 | 7.5 | 300.0 | Human frataxin |
| 4985 | native | 1101 | 0.0244 | 0.00152 | 7.0 | 303.0 | Flavodoxin-like domain of E. coli sulfite reductase |
| 2956 | unknown | 142 | 0.0189 | 0.01056 | 6.0 | 295.0 | lac repressor headpiece |
| 5022 | native | 611 | 0.0179 | 0.00283 | 6.0 | 303.0 | UmuD2C |
| 6970 | native | 721 | 0.0171 | 0.00049 | 6.8 | 298.0 | Small Rho-GTPase Rac1 |
| 11338 | native | 833 | 0.0164 | 0.00072 | 7.0 | 296.0 | Human RWD domain containing protein 1 |
| 6047 | native | 1128 | 0.0161 | 0.00066 | 7.5 | 300.0 | Organomercurial lyase MerB |
| 15855 | native | 1012 | 0.0127 | 0.00195 | 7.4 | 298.0 | Human Growth Arrest and DNA Damage alpha protein (Gadd45a) |
| 10010 | native | 757 | 0.0127 | 0.00045 | 6.5 | 313.0 | Bovine beta-lactoglobulin A34C mutant |
| 5070 | native | 636 | 0.0100 | 0.00058 | 6.8 | 308.0 | Bacillus stearothermophilus tyrosyl-tRNA synthetase (C-term) |

^a^intrinsically disordered

^b^partially folded

^c^molten globule

^d^state not provided
